# Improving growth and yield of *Cucurbita argyrosperma* with goat manure

**DOI:** 10.3389/fpls.2025.1658365

**Published:** 2025-12-02

**Authors:** Nomfanelo Zondo, Nontuthuko R. Ntuli, Sydney Mavengahama, Corlina M. Van Jaarsveld

**Affiliations:** 1 Department of Agriculture, Faculty of Science, Agriculture and Engineering, University of Zululand, KwaDlangezwa, South Africa; 2 Department of Botany, Faculty of Science, Agriculture and Engineering, University of Zululand, KwaDlangezwa, South Africa; 3 Food Security and Safety Niche Area, Faculty of Natural and Agricultural Sciences, North-West University Mmabatho, Mmabatho, South Africa

**Keywords:** food security, Japanese pie pumpkin, neglected and underutilized crop, organic fertilizer, traditional leafy vegetable

## Abstract

*Cucurbita argyrosperma* Huber is a traditional leafy vegetable crop where different plant parts are consumed by rural communities in South Africa especially in the northern KwaZulu-Natal, South Africa. Goat manure is a nutrient-rich organic fertilizer that is readily available in rural communities of KwaZulu-Natal. Little is known about the organic fertilizer requirements of *C. argyrosperma*. Therefore, this study aimed to determine the effect of goat manure application on the growth and yield of *C. argyrosperma*. A field experiment was conducted at the University of Zululand farm in northern KwaZulu-Natal in which *C. argyrosperma* was grown under six goat manure application rates (0, 5, 10, 15, 20 and 25 t ha^–1^) over two seasons in a randomized complete block design with four replications. Data were collected on vegetative growth traits (vine length, stem diameter, leaf area, number of branches and leaves, and leaf chlorophyll content), as well as marketable yield (shoot, fruit and seed fresh mass, shoot dry mass, and fruit and seed number). An analysis of variance test was performed on the pooled data to identify significant differences at p < 0.05 and the means were separated using Tukey’s test. Plants treated with 20 and 25 t ha^–1^ manure had significantly (p < 0.05) more branches and leaves, longer and heavier harvested shoots and a higher fruit number and mass and 100-seed mass than control. The remaining parameters increased with an increase in goat manure application, but not significantly. The yields obtained in this study were relatively low compared to typical yields for this crop and require further investigation. This study showed goat manure application could improve growth and yield of *C. argyrosperma* and ultimately contribute to food security in rural communities at affordable agronomic inputs.

## 1 Introduction

*Cucurbita argyrosperma* Huber commonly known as cushaw pumpkin belongs to the family Cucurbitaceae and is an annual herbaceous climber (Najera et al., 2018). Its stems grow as long vines (Balvino-Olvera et al., 2017) and are sparsely branched with soft whitish hairs (Ntuli et al., 2016). The leaves are slightly lobed (Jones, 1993) and green in color with soft spines (Ntuli et al., 2016). *Cucurbita argyrosperma* has monoecious flowers (Balvino-Olvera et al., 2017), that develops into grayish-green fruits and flattened white seeds (Ntuli et al., 2016; Najera et al., 2018). It is a traditional leafy vegetable (TLV) that is commonly consumed by rural communities in the northern part of KwaZulu-Natal, South Africa (Ntuli et al., 2017). The leaves, flowers, fruits, and seeds of this plant are eaten (Van Rensburg et al., 2007).

Traditional leafy vegetables (TLVs) are wild or semi-domesticated plants that are rich in nutrients and well adapted to the local climate (Njume et al., 2014) and have the potential to contribute to household food security in rural communities (Mudau et al., 2022; Qwabe and Pittawaty, 2022; Zulu et al., 2022). These communities often face unemployment, lack of resources and food insecurity in South Africa (Shackleton et al., 2008; Zulu et al., 2022). Increased agricultural activities have been suggested as a possible means to increase food production in these areas (Mudau et al., 2022; Qwabe and Pittawaty, 2022; Zulu et al., 2022). However, the utilization of TLVs has drastically declined over the years (Odhav et al., 2007; Mathaba, 2017), because people prefer commercialized vegetables (Lewu and Mavengahama, 2011; Mathaba, 2017).

South Africa has a goat population of more than 35 million animals that are mostly kept in rural areas where they form an important part of the local culture. Goats are relatively easy and inexpensive to keep (Mataveia et al., 2021). In Sub-Saharan Africa, unused goat manure often accumulates in large quantities which is an environmental concern (Washaya and Washaya, 2023). Goat manure is an organic fertilizer that contains sufficient nitrogen (N), phosphorus (P) and potassium (K) for the growth of most crops and is particularly rich in N and P compared to most organic fertilizers (Wuta and Nyamugafata, 2012). It typically contains 2.2 – 2.6% N, 0.2 – 0.9% P and 2.2 – 4.1% K (Younis et al., 2000). Goat manure is an attractive alternative to rural farmers who cannot afford inorganic fertilizers (Law-Ogbomo and Osaigbovo, 2018). In addition, soils in KwaZulu-Natal are often low in nutrients and the application of fertilizer is thus critical (Mbatha et al., 2021). As an organic fertilizer, goat manure is not only a source of nutrients but serves as a soil amendment by improving the physical properties of soils (Budiyanto et al., 2022).

Application of goat manure in cucumber (*Cucumis sativus)* significantly increases vine length, number of branches and leaves, fruit length, fruit girth, number of fruits per plant, and fruit yield (Law-Ogbomo and Osaigbovo, 2018). In the fluted pumpkin (*Telfairia occidentalis*), goat manure application increases the leaf area by producing broader leaves (Umekwe et al., 2020). Likewise, fruit mass and diameter, and thickness of the edible fruit part of the melon (*Cucumis melo*) are increased by goat manure application (Handajaningsih et al., 2018). Limited studies on organic cropping were previously conducted on cucurbits but not on the effect of goat manure on *C. argyrosperma*. Knowing the optimum fertilizer application rate of a crop is one of the most fundamental agronomic requirements for its successful cultivation. Therefore, this study aimed to determine the effect of goat manure on the growth and yield of *C. argyrosperma*.

## 2 Materials and methods

### 2.1 Experimental site, treatments, and experimental layout

The study was conducted at the University of Zululand farm situated at KwaDlangezwa (Empangeni) in northern KwaZulu-Natal, South Africa (28° 51’ 0” S and 31° 49’ 60” E). The experiment tested six goat manure treatments applied at 0, 5, 10, 15, 20, and 25 t ha^–1^ which were replicated four times in a randomized complete block design. Each plot was 8 m x 5 m consisting of five rows with a 1 m x 1 m intra- and inter-row spacing, as well as inter-plot spacing of 2 m. Seven plants were planted per row. The experiment was conducted over two seasons (2020 and 2021) (**Table 1**).

**Table 1:** Data of weather recorded during the first and the second trial of the experiment.

Season	Month	T_min_	T_max_	Heat units	RHP	Rain	SRAD
2020	August	12.6	24.9	8.75	52	32.2	14
	September	15.3	26.5	10.9	58	69.8	15.3
	October	17.7	27.3	12.5	64	68.9	17.4
	November	19.2	29	14.1	65	77.2	19.9
	December	20.8	31.3	16.05	64	87.7	21.4
	Total	–	–	62.30	–	335.80	–
	Mean	17.12	27.80	–	60.60	67.16	17.60
2021	August	19.90	29.52	14.71	66.87	31.8	19.57
	September	15.17	26.12	10.65	58.60	134.6	16.30
	October	15.64	25.95	10.80	67.39	102.7	16.13
	November	18.07	27.60	12.84	65.03	43.0	19.54
	December	20.12	29.48	14.80	68.00	194.9	18.88
	Total	–	–	63.80	–	507	–
	Mean	17.78	27.73	–	65.18	101.40	18.08

Tmin, minimum temperature (°C); Tmax, maximum temperature (°C); RHP, relative humidity percentage at 14 h00 (%); Rain, (mm); SRAD, solar radiation (MJ/m^2^/d).

### 2.2 Soil and compost analysis

Analysis of the composite soil and compost samples collected before the experiment was done according to Manson et al. (2020) (**Table 2**). The pH (KCl) was measured using 1 M KCl-solution with gel-filled combination glass electrode. The extractable phosphorus, potassium, zinc, manganese and copper of the soil were determined using a Ambic-2 extraction. The phosphorus was measured using the Murphy and Riley (1962) molybdenum blue procedure (Hunter, 1974). Potassium, zinc, copper and manganese were determined by atomic absorption. The extractable calcium, magnesium and acidity of the soil were obtained from a 1 M KCl extraction, and the Ca and Mg content was measured with atomic absorption. The total cations (CEC) were calculated as the sum of KCl-extractable Ca, Mg, and acidity and Ambic-2 extractable K and the acid saturation was calculated from the extractable acidity. The organic carbon, total nitrogen content and clay content of the soil were estimated by mid-infrared reflectance (with a Bruker Tensor 27) using the air-dry, milled soil samples. The extraction of minerals (P, K, Ca, Mg, Na, Cu, Zn, Fe, Mn and Al) in the compost was by 1 M HCl and the concentration of the minerals was measured with ICP-OES (inductively coupled plasma optical emission spectroscopy). An automated Dumas dry combustion method using a LECO TruMac CNS (LECO Corporation, Michigan, USA; Matejovic, 1996) was used for the total nitrogen and carbon content of the compost.

**Table 2:** Physico-chemical analysis of the soil and goat manure (GM) used in both experiments.

Characteristics	Season 2020		Season 2021	
	Soil	GM	Soil	GM
N (%)	0.07	3.03	0.05	2.12
P (mg/kg)	5.21	0.72	12.90	0.65
K (mg/kg)	113,54	2.48	44.35	0.97
Calcium (Ca) (mg/kg)	483.33	2.83	319.35	2.38
Magnesium (Mg) (mg/kg)	178.13	1.01	78.23	0.82
Aluminium (Al) (mg/kg)	–	9985	–	16514
Copper (Cu) (mg/kg)	3.02	46.2	0.97	63.0
Iron (Fe) (mg/kg)	–	17085	–	27172
Manganese (Mn) (mg/kg)	2.08	565	4.03	817
Sodium (Na) (mg/kg)	–	5952.2	–	1423.2
Zinc (Zn) (mg/kg)	1.25	246	3.06	187
Organic carbon (%)	1.0	29.66	1.1	21.00
pH (KCl)	4.36	7.19	4.31	8.32
Moisture (%)	–	59.77	–	51.53
Exchangeable Acidity (cmol/kg)	0.35	–	0.19	–
Total cations (cmol/kg)	4.52	–	2.55	–
Acid saturation (%)	8	–	8	–
Clay (%)	45	–	16	–

### 2.3 Crop management

Weather data for the study area were collected from The South African Sugarcane Research Institute’s Empangeni weather station for the duration of the experiment (**Table 1**). Both the temperature and rainfall were higher in season two than in season one. Prior to planting, a composite soil sample was collected in a zig-zag pattern from the experimental site to a depth of 20 cm with a soil auger, bulked, and analyzed for physico-chemical properties (**Table 2**). The land was ploughed and disked to a fine tilth.

Goat manure was broadcasted and worked into the soil 14 days before planting. Seeds were collected from the uMkhanyakude local community (Mseleni: 27° 38” S, 32° 47” E). A maximum of five fruits had their seeds mixed prior to sowing to eliminate biases in germination cues. The seeds were dried in a cool and dry place with adequate ventilation. The dried seeds were then stored in an air-tight container, until the commencement of the trial. Three *C. argyrosperma* seeds were planted in holes to a depth of 5–8 cm (Paulauskiene et al., 2018), which was thinned to one seedling per stand two weeks after planting (Oloyede et al., 2014). Plants were rainfed throughout the growing season. Weeding was performed when necessary. Pruning of the apical bud was done using secateurs on all stems during the vegetative stage once the first three true leaves were fully expanded. The pruning was done once at 27 days after sowing (DAS) to stimulate branching.

### 2.4 Data collection

Both the vegetative and reproductive morphological traits were determined according to Okporie et al. (2012). Five plants were randomly selected from the net plot (inner rows of each plot) and marked for measuring vegetative traits. The marked plants were used to determine all morpho-agronomic traits.

#### 2.4.1 Vegetative traits

Vine length (from base to the tip; in cm) was determined from the longest vine using a measuring tape, at 47 DAS, only in season one, before harvesting the three leaf shoot tips. This was not repeated in season two to minimize fruit abortion that is caused by frequent touching of the reproducing vines which happened in season one. Stem diameter, leaf area, and leaf chlorophyll content were determined at 47 DAS for both seasons when vegetative growth was sufficient for harvesting. The stem diameter (cm) of the main stem was measured at 2 cm from the soil level using a Vernier calliper. The number of branches and leaves per plant were counted manually at 47 DAS. The second fully expanded leaf of each shoot tip was used to measure the length (L; in cm) and width (W; in cm) of the leaf. The leaf area was calculated, as determined for cucumber leaves, as follows:


$$Leaf\;Area(LA;\;cm^{2})=0.88(L\times W)$$

(Blanco and Folegatti, 2005)

The leaf chlorophyll content (nmol m^−2^) was measured using a portable chlorophyll meter (CCM- 200 PLUS, Opti-Sciences, USA). The measurements were done on the fifth fully expanded leaf from the apex with three readings per leaf.

#### 2.4.2 Marketable yield

On day 88 after sowing in season one and two, three leaf shoot tips were harvested from all the branches of plants of the net plot (inner rows). On the same day, the shoot length of each harvested shoot tip was measured. The harvested shoot tips were weighed for fresh and dry mass. The fresh mass of *C. argyrosperma* shoot tips was determined immediately after harvest to avoid water loss using a Metler balance (Model PN 163). The dry mass of the three leaf shoot tips was obtained by drying the samples in an oven (Baird and Tatlock, Greenfield, England) at 80 °C until stable mass was obtained. The marketable yield of fruits (uniform color, size, and shape, undamaged by pests and disease free) was separated from the unmarketable fruits and counted per net plot (inner rows). The fresh mass of the harvested fruit was determined using a Metler balance (Model PN 163) and was extrapolated to kg ha^–1^. The marketable yield of seeds was determined by extracting seeds from the mature fruits of each plot prior to drying the fruit. The extracted seeds were dried in a cool and dry place with adequate ventilation, and the total number of seeds per fruit was counted. Total seed mass (kg ha^–1^) and the 100-seed mass (g) were determined using a Metler balance (Model PN 163).

### 2.5 Data analysis

The data were pooled for the two seasons. The recorded data were subjected to analyses of variance (ANOVA) using GenStat^®^ Version 12 (Susanto et al., 2013). Where the F-test showed significant differences, Tukey’s test was used to separate the means at 5% significance level.

## 3 Results

None of the differences in vine length, stem diameter, leaf area and leaf chlorophyll content among manure application treatments were significant (**Table 3**). However, the general trend was for values to increase with an increase in manure application rate. Differences in branch and leaf number were significant (p < 0.05) with the 20 t ha^−1^ goat manure treatment having a higher branch number than all the other treatments. The leaf number of the 25 t ha^−1^ manure treatment was higher than the 0, 10 and 15 ha^−1^ manure treatments.

**Table 3:** Effect of goat manure application on the vegetative traits of *Cucurbita argyrosperma* at 47 days after sowing.

Manure application rate (t ha^–1^)	Branch number	Vine length (mm)	Stem diameter (mm)	Leaf area (cm^2^)	Leaf number	Leaf chlorophyll content (g m^–2^)
0	1.54^b^	426	3.6	91	22.4^c^	20.8
5	1.36^b^	869	4.5	100	34.6^ab^	21.1
10	1.45^b^	680	5.0	142	28.8^c^	24.6
15	1.31^b^	792	6.1	158	30.8^bc^	24.2
20	2.28^a^	952	5.0	124	37.1^ab^	22.7
25	1.71^ab^	1145	6.8	227	39.5^a^	25.7
Significance	p < 0.001	p = 0.717	p = 0.153	p = 0.081	p < 0.001	p = 0.132
HSD_0.05_	0.4	ns	ns	ns	5.4	ns

For each trait, means with different letter(s) within rows differ significantly at p < 0.05. ns, not significant.

The shoot length, fresh and dry shoot mass of *Cucurbita argyrosperma* plants were significantly (p < 0.05) affected by manure application (**Table 4**). The shoot length of the harvested vines was longer in plants treated with 10, 15, 20 and 25 t ha^−1^ manure than 5 t ha^−1^ manure, which, in turn, was longer than the control plants. The shoot fresh and dry mass was the highest of all the treatments at 20 and 25 t ha^−1^ goat manure application.

**Table 4:** Effect of goat manure application on *Cucurbita argyrosperma* shoot length and fresh and dry shoot mass of harvested shoot tips.

Manure application rate (t ha^–1^)	Shoot length (mm)	Shoot fresh mass (kg ha^–1^)	Shoot dry mass (kg ha^–1^)
0	337.8^c^	189.8^c^	26.8^b^
5	447.8^b^	235.4^b^	30.1^b^
10	545.2^a^	250.9^b^	31.9^b^
15	553.1^a^	273.6^b^	36.5^b^
20	555.3^a^	327.7^a^	50.0^a^
25	583.1^a^	356.7^a^	57.5^a^
Significance	p < 0.001	p < 0.001	p < 0.001
HSD_0.05_	39.0	27.3	6.8

For each trait, means with different letter(s) within rows differ significantly at *p* < 0.05. ns, not significant.

Applying 20 and 25 t ha^−1^ manure produced a higher fruit number, fruit mass, and 100-seed mass when compared with 0, 5, and 10 t ha^−1^ treatments (**Table 5**). Also, the 20 t ha^−1^ manure application treatment had higher values than the 25 t ha^−1^ manure application treatment for the 100-seed mass. The application of goat manure had no effect on the number of seeds per fruit or the seed mass per hectare, but they did show a general increase with an increase in manure application rate.

**Table 5:** Fruit and seed yield traits of *Cucurbita argyrosperma* in response to different rates of goat manure application.

Manure application rate (t ha^–1^)	NF (ha^–1^)	FM (g fruit^1^)	NOS (fruit ^-1^)	TSM (kg ha^-1^)	100-SM (g)
0	679^c^	169^c^	108	13.6	12.1^d^
5	1220^bc^	317^c^	118	41.0	13.4^cd^
10	502^c^	130^c^	135	33.5	14.1^c^
15	1859^b^	708^b^	150	71.1	14.7^c^
20	2810^a^	1170^a^	155	86.5	19.0^a^
25	3500^a^	958^ab^	178	113.9	16.8^b^
Significance	p < 0.001	p < 0.001	p = 0.462	p = 68.1	p < 0.001
HSD_0.05_	574.6	251.5	ns	ns	1.3

For each trait, means with different letter(s) within columns differ significantly at p < 0.05. NF, number of fruits; FM, fruit mass; NOS, number of seeds; TSM, total seed mass; 100-SM, 100-seed mass; HSD, honest significant difference.

## 4 Discussion

### 4.1 Effect of manure on vegetative traits

There was in increase in *C. argyrosperma* vine length, stem thickness, leaf size and leaf chlorophyll content with an increase in goat manure application, although the differences were insignificant. Branch and leaf number were highest at 20 and 25 t ha^−1^ goat manure (**Table 3**). Vegetative growth in plants is promoted by nitrogen (Shorna et al., 2020; Kakoli et al., 2025). Nitrogen is of particular importance in vegetables and is also associated with the production of fruits and seeds (Mohammed et al., 2021).

A study on *Solanum nigrum* where goat manure was applied at rate of about 200 kg N ha^−1^ (compared to 128 to 625 kg N ha^−1^ in the current study) also recorded a non-significant effect on plant height, stem diameter, leaf area and leaf chlorophyll content (Bvenura and Afolayan, 2013). Similarly, goat manure applied at rate of 178 kg ha^−1^ N did not significantly increase plant height and number of leaves in zucchini (*Cucurbita pepo*) (Subedi et al., 2024). The insignificant responses to manure could be linked to the slow release of organic fertilizers compared to inorganic fertilizers (Mhlontlo et al., 2007; Geng et al., 2019). Another study on *C. pepo* showed that applying compost, vermicompost and dry *Azolla* fertilizer at rates varying from 113 to 117 kg ha^−1^ N, only increased leaf chlorophyll content under the dry Azolla fertilizer (Youssef et al., 2021). In *C. moschata*, applying cow manure at 30 t ha^-1^ increased plant height, leaf number and branch number relative to the lowest inorganic fertilizer application treatment (Kakoli et al., 2025). An increase in leaf number and leaf area in crops is important in increasing photosynthesis which, in turn, may increase yield (Bako et al., 2021). The goat manure used in this study was also a source of phosphorus (**Table 2**). A study on cucumber (*Cucumis sativus*) showed that phosphorus increased the number of branches (Adediran et al., 2014) and could explain the observed increase in branch number in the current study.

Mbhele and Ntuli (2019) reported that applying inorganic fertilizer (NPK and limestone ammonium nitrate) at total N application of 104 kg ha^-1^ resulted in longer vines and thicker stems in *C. argyrosperma*. This study also showed that applying basal N on its own without topdressing with limestone ammonium nitrate did not affect the leaf chlorophyll content. Leaf chlorophyll content generally corresponds with leaf nitrogen content which induces the chlorophyll biosynthesis processes (Geremew et al., 2021) and is an indicator of the health status of plants (Bvenura and Afolayan, 2013). It was reported that organic fertilizers do not always result in an increase in leaf chlorophyll content, because of their relatively low nitrogen content and slow release of nutrients (Adekiya et al., 2020).

### 4.2 Marketable yield

In the present study, the application of 10, 15, 20 and 25 t ha^–1^ of manure resulted in higher shoot length, and fresh and dry shoot mass than in the control plants (**Table 4**). Similarly, in a study on the fluted pumpkin (*Telfaria occidentalis*), the application of 25 t ha^–1^ of a goat manure (composition not provided) significantly improved harvestable (leaf) yield (Umekwe et al., 2020). The application of inorganic N-containing fertilizers at different rates had an inconsistent effect on the shoot yield in *C. argyrosperma* (Mbhele and Ntuli, 2019). The higher marketable fresh and dry mass at 20 and/or 25 t ha^−1^ manure application in the current study may be explained by the higher number or branches and leaves and longer harvested shoots observed for these treatments.

With the exception of seed mass and number, all the fruit and seed yield parameters (number of fruits, fruit mass, number of seeds, and 100-seed mass) were higher in plants treated with 20 and 25 t ha^–1^ manure (containing about 500 to 600 kg ha^−1^ N) when compared with the control plants (**Table 5**). The result of the present study concurs with Jahan et al. (2013) who reported that the optimum cattle manure application for fruit yield of *C. pepo* was 20 t ha^−1^ (422 kg ha^−1^ N). Also, applying cattle manure did not significantly affect the seed yield in their study. Applying composted chicken manure at 2.5 to 10 t ha^−1^ (67 to 267 kg ha^−1^ N) and a leachate to soil or leaves increased seed mass, but not fruit mass in *C. argyrosperma* (Rodríguez-Cuevas et al., 2025). In addition, *C. argyrosperma* plants treated with inorganic N-containing fertilizer at a rate of 104 kg ha^−1^ N resulted in the highest fruit number and size, but did not increase the seed yield (Mbhele and Ntuli, 2019).

Despite the beneficial effects of goat manure in the present study, the fruit and seed yield were low compared to other studies. For example, the maximum fruit numbers varied from 3015 to 8983 ha^−1^ in various studies (Jahan et al., 2013; Mbhele and Ntuli, 2019; Ntuli et al., 2017) compared to the maximum of 3500 ha^−1^ in the current study. Likewise, the highest fruit mass in the current study was 1170 g but ranged from 900 to 1690 in similar research (Jahan et al., 2013; Mbhele and Ntuli, 2019; Rodríguez-Cuevas et al., 2025). The maximum number of seeds per fruit was 302 (Mbhele and Ntuli, 2019) and 623 (Jahan et al., 2013) but only 178 in this study. However, the 100-seed mass of the current study (maximum 16.8 g) corresponded well with other studies. Ntuli and Mbhele (2019) reported a maximum 100-seed mass of 15.7 g, while Jahan et al. (2013) reported 0.13 g. The low yield could be the result of various climatic, cultural, soil or genetic factors (Liliane and Mutengwa, 2020) and needs further investigation. For example, pumpkin yield was significantly affected by rainfall, temperature, and cultivar (Da Silva et al., 2025) and by sowing date and plant density (Wadas and Kalinowski, 2010).

Applying a commercial organic fertilizer was able to improve the bulk density and water use efficiency of the soil and to increase the soil nutrient (organic C, N, P and K) content. This in turn increased the stem thickness, vine length, and leaf area in pumpkin (*Cucurbita* variety) and ultimately its fruit yield (Ren et al., 2024). The link between the physical, chemical and biological benefits of organic fertilizers and their ability to improve growth and yield in cucurbits is also supported by other studies (for example Khandaker et al., 2022; Yekula et al., 2023; Subedi et al., 2024; Tian et al., 2025). This link may also explain the observed increase in growth and yield in *C. argyrosperma* due to the application of goat manure in the current study.

## 5 Conclusion

The growth (leaf and branch number) and yield (shoot length and mass, fruit number and mass and 100-seed mass) of *Cucurbita argyrosperma* increased with an increase in goat manure applications up to 25 t ha^−1^. This outcome underlines the potential of goat manure as suitable fertilizer to successfully grow *C. argyrosperma.* However, the yields obtained in this study were low and require further investigation.

## Data Availability

The raw data supporting the conclusions of this article will be made available by the authors, without undue reservation.
